# Yeast to Study Human Purine Metabolism Diseases

**DOI:** 10.3390/cells8010067

**Published:** 2019-01-17

**Authors:** Bertrand Daignan-Fornier, Benoît Pinson

**Affiliations:** 1 Université de Bordeaux IBGC UMR 5095 1, rue Camille Saint-Saëns, F-33077 Bordeaux, France; benoit.pinson@ibgc.cnrs.fr; 2 Centre National de la Recherche Scientifique IBGC UMR 5095 1, rue Camille Saint-Saëns, F-33077 Bordeaux, France

**Keywords:** purine metabolism, nucleotide synthesis, purine-associated deficiencies, hyperuricemia, Lesch–Nyhan, AMP-deaminase, ATIC, ADSL, PRPS

## Abstract

Purine nucleotides are involved in a multitude of cellular processes, and the dysfunction of purine metabolism has drastic physiological and pathological consequences. Accordingly, several genetic disorders associated with defective purine metabolism have been reported. The etiology of these diseases is poorly understood and simple model organisms, such as yeast, have proved valuable to provide a more comprehensive view of the metabolic consequences caused by the identified mutations. In this review, we present results obtained with the yeast *Saccharomyces cerevisiae* to exemplify how a eukaryotic unicellular organism can offer highly relevant information for identifying the molecular basis of complex human diseases. Overall, purine metabolism illustrates a remarkable conservation of genes, functions and phenotypes between humans and yeast.

## 1. Introduction

Purine nucleotides, adenosine 5’-triphosphate (ATP), guanosine 5’-triphosphate (GTP) and their derivatives are involved in a myriad of cellular processes: energy storage, synthesis of nucleic acids and coenzymes (Nicotinamide adenine dinucleotide (NAD)/Nicotinamide adenine dinucleotide phosphate (NADP)/coenzyme A/flavine adenine dinucleotide (FAD)), translation, signaling, etc. These molecules are thus absolutely required for all known forms of life, and their synthesis results essentially from conserved pathways. In yeast, mutations abolishing ATP or GTP synthesis are lethal, although lethality may require more than one mutation due to genetic and pathway redundancy [1]. Even a partial block of purine metabolism can have drastic physiological consequences, and several diseases associated with purine metabolism dysfunctions have been reported in human [2,3,4,5]. Some purine metabolic disorders have been described for a long time, such as hyperuricemia (gout), which is caused by an excess of uric acid (the final purine degradation product) leading to a painful deposit of urate crystals in joints. Among the studies of genetic alterations leading to hyperuricemia, hypoxanthine phosphoribosyl transferase (HGPRT)-deficiency, involved in the Lesch–Nyhan syndrome, was one of the very first genetic-disease enzymes identified in humans [6]. In addition to hyperuricemia, purine metabolism-associated diseases share a large spectrum of immunological, hematological and neuro-muscular disorders [7], and are all characterized by an abnormal level of purine nucleotides in cells and of nucleosides and/or nucleobases in bodily fluids [8]. In most cases, the dysfunctional gene in purine metabolism is known. However, this identification of the causative enzyme does not necessarily give clear indications on the etiology of the disease and hence, a more comprehensive view of the metabolic consequences of the dysfunction is often needed. To this end, model organisms that are amenable to genetics can be valuable for identifying critical functions that are affected as a consequence of the primary metabolic dysfunction. Several animal or microbial models can be used for this purpose and are often highly complementary. In this review, we present results obtained with the budding yeast, *Saccharomyces cerevisiae*, to illustrate how a unicellular eukaryotic organism can offer highly relevant information to help understand the mechanisms leading to complex human diseases.

Yeast can be both a source of information and a tool used to study human metabolic diseases. In both cases, the relevance of the information drawn from yeast depends on the functional conservation between yeast and humans, a conservation which is often much higher than one could have initially thought based on their long divergent evolution (over one billion years). In a systematic replacement of yeast genes by their human orthologues, Marcotte and coworkers showed that nearly half of the yeast genes could be successfully “humanized” [9], thus confirming the wide potential of yeast as a model to study diseases associated to human gene dysfunctions. As a unicellular eukaryotic organism yeast is a very polyvalent tool, since it is highly amenable to molecular genetics but also to analytical biochemistry. Studies on yeast have provided a multitude of information on metabolic pathways and how they are connected with one another as well as to other cellular functions. In addition, as an organism facing nutrient changeability and stress [1], it also delivered key information on how metabolic homeostasis is achieved in complex systems. This integrated view offered by yeast research is of great value to help address complex metabolic diseases in human. Indeed, most diseases are the result of multiple effects and take place in a highly integrated genetic and physiological context. Knowledge collected from yeast can be used to generate testable hypotheses in human cells or model animals. 

In this review, we will focus on purine metabolic diseases and illustrate how yeast can be used to help promote our understanding of the mechanisms involved. As stated above, yeast can be used as a model provided that sufficient functional conservation is found. However, to be highly informative as a model, yeast should demonstrate both conservation of enzymes and pathways, as well as a conserved functional organization including interactions between the metabolic pathways. This last aspect cannot be generalized and requires thorough specific investigations. Several examples developed below illustrate the remarkable level of conservation in the “logic” of metabolism and in the consequences of its dysfunctions. This suggests that the general mechanisms responsible for nucleotide homeostasis are very ancient and that their conservation has been under high selective pressure.

## 2. Purine Metabolism in Human and Yeast: Similarities and Differences

### Purine Metabolic Pathways: Functions Are Generally Conserved but Protein Sequences Can Diverge

**Figure 1:**
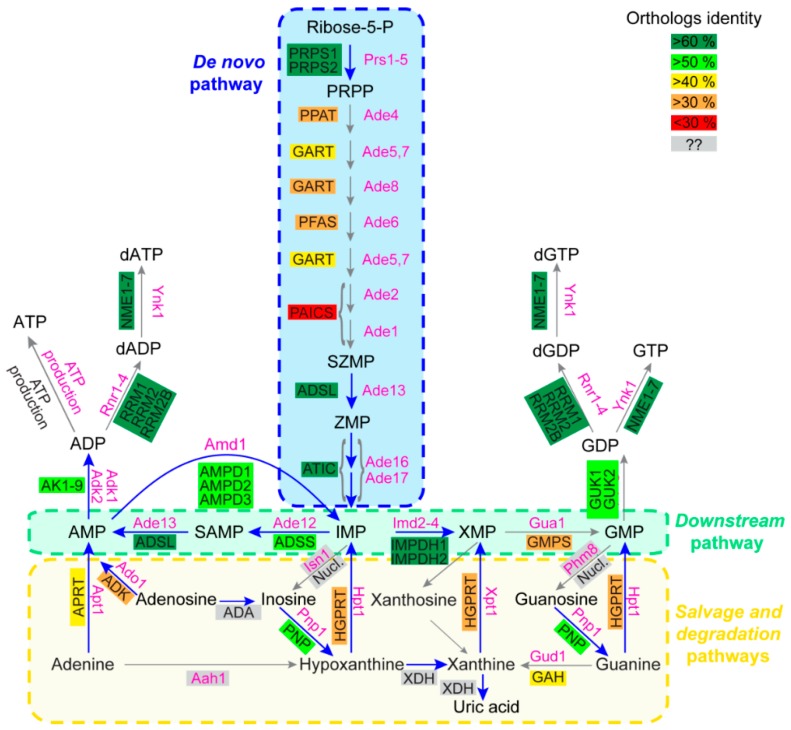
Schematic representation of the human and *Saccharomyces cerevisiae* purine biosynthesis pathways. Features of human and yeast enzymes are listed in Table A1, Table A2 and are shown in black and pink, respectively. For human enzymes, the numerous isoforms detected for some enzymes are not presented. Ortholog identity scores were determined as described in supplementary Table A3. Grey boxes correspond to enzymes with no or unidentified orthologs between yeast and human. “ATP production” in both yeast and human cells corresponds to the different ATP production pathways such as glycolysis and the respiratory chain/ATP synthase complexes. “Nucl.” stands for the numerous human nucleotidases, such as for example the NT5 family. Abbreviations: IMP: Inosine monophosphate; PRPP: Phosphorybosyl pyrophosphate; SAMP: Succinyl-AMP; SZMP: Succinyl Amino Imidazole Carboxamide Ribonucleotide monophosphate; XMP: Xanthosine monophosphate; ZMP: Amino Imidazole CarboxAmide Ribonucleotide monophosphate.

Most enzymatic steps of the purine de novo and recycling pathways are catalyzed by proteins that are largely conserved between prokaryotes and eukaryotes [1]. Accordingly, a high degree of conservation is found between yeast and human purine pathways. The ten enzymatic steps of the de novo pathway are fully conserved, although the percentage of identity among orthologous enzymes is variable, as illustrated in Figure 1. Remarkably, the four most conserved proteins (>60% identity between orthologs) in the de novo and downstream pathways are mutated in reported diseases (Figure 1, blue arrows), i.e., Phosphoribosyl pyrophosphate (PRPP) synthetase (PRPS1); adenylosuccinate lyase (ADSL), aminoimidazole carboxamide riboside monophosphate transformylase inosine 5’-monophosphate cyclohydrolase (ATIC) and IMP dehydrogenase (IMPDH) (Table 1). Of note, both the ADSL and ATIC steps metabolize intermediates acting as regulators of various functions in both yeast and humans (see below, Section 3.2) [10,11,12,13,14]. A lower identity is detected for proteins catalyzing the seven other steps of the de novo pathway, and several of these enzymes are encoded as gene fusions in the human genome (see GART and PAICS, Figure 1). For the purine salvage pathway, conservation varies between the nucleotide monophosphate interconversion enzymes and the nucleoside/nucleobase salvagers. Indeed, the interconversion of monophosphate nucleotides is ensured by highly conserved proteins, while enzymes metabolizing nucleosides and nucleobases show little to no conservation (Figure 1). Among those enzymes, the first group corresponds to proteins that are highly divergent in sequence but for which catalytic activity is nevertheless conserved. These proteins correspond to the phosphoribosyl transferases (APRT and HGPRT, Figure 1), which catalyze nucleotide monophosphate synthesis from nucleobases, and to the nucleosidases (Phm8 [15] and Isn1 [16] in yeast and the NT5 nucleotidase family in human [17]) that catabolize nucleotide monophosphate to nucleosides. The second group contains proteins with no orthologs between yeast and humans. For example, in humans, adenosine is deaminated into inosine by ADA [18] with no yeast ortholog and, by contrast, the yeast adenine deaminase (Aah1) catalyzing adenine to hypoxanthine conversion [19] has no ortholog in human cells (Figure 1). Of note, this difference in the evolution of nucleobase and nucleoside metabolism in yeast versus humans is strengthened by the fact that nucleobases are the only purine precursors taken up by yeast [1] via the concentrative carrier Fcy2 [20], while nucleobases and nucleosides are both transported into human cells by a set of equilibrative (hENT) and concentrative (hCNT) membrane transporters [21]. Another major difference observed between yeast and humans relates to purine degradation. In humans, nucleobases are transformed to uric acid by xanthine oxidoreductases [8], while in yeast, removal of purine excess is performed by nucleobase excretion (essentially hypoxanthine) [13,22,23]. Indeed, no xanthine oxidase activity has been reported in yeast. By contrast, the other purine degradation steps which are common to both yeast and humans correspond to purine nucleoside phosphorylase (inosine to hypoxanthine transformation by PNP/Pnp1) and guanine deaminase (guanine to xanthine metabolization by GAH/Gud1), which are both well conserved between yeast and humans (Figure 1). 

**Figure 2:**
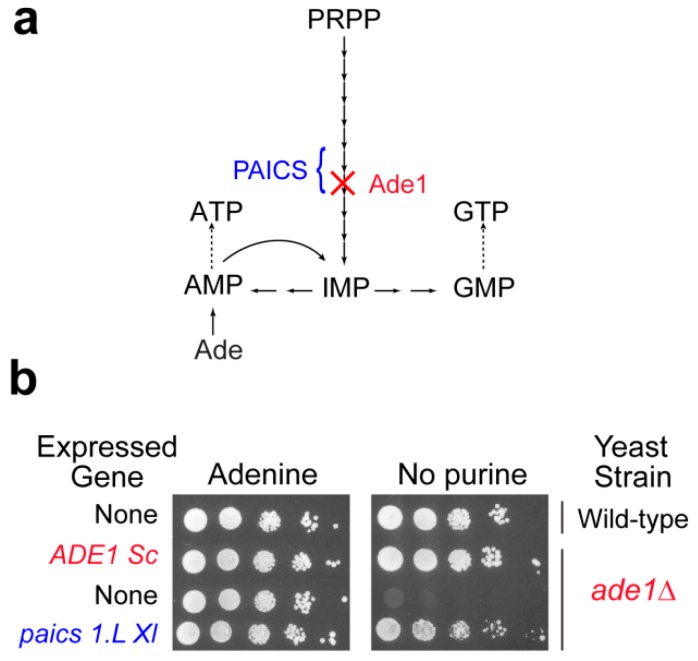
Functional complementation of the yeast phosphoribosyl aminoimidazole succinocarboxamide synthetase by the *Xenopus laevis* ortholog *paics1.L*. (**a**). Simplified scheme of the yeast purine pathway. (**b**). Yeast wild-type and knock-out mutant (*ade1*Δ) strains were either transformed with a plasmid allowing expression of the yeast (*ADE1* Sc) or the *Xenopus laevis* (*paics 1.L*) amino imidazole succinocarboxamide synthetase encoded gene, or with the empty vector (None). Serial dilutions (1/10) of transformants were dropped on SDCASAW medium to score the ability of the yeast and xenopus genes to complement the *ade1* mutant auxotrophy observed in the absence of external purine source. A medium supplemented with adenine was used as a viability control of transformants and images were obtained after 34 h of growth at 37 °C.

In summary, this comparison highlights that the purine-associated human diseases correspond to alterations in the most conserved steps of the purine pathways (Figure 1, blue arrows). When altered, these steps are also detrimental in yeast and belong to the following pathways: (1) The monophosphate interconversion pathway which is common to the de novo and salvage pathways (downstream pathway), which allows interconversion between purines and the balanced synthesis of the final products ATP and GTP (Figure 1), (2) the phosphoribosyl pyrophosphate (PRPP) synthesis which is required for both the de novo pathway (for PPAT/Ade4) and the salvage pathway (for the phosphoribosyl transferases APRT and HGPRT) and (3) purine degradation (via PNP/Pnp1). In most of these purine-associated diseases, however, it is not clear whether the detrimental effects are linked to a toxic accumulation of the substrate(s) of these enzymes, to a lack of their products, or to a combination of both effects. The high degree of conservation observed between the two organisms allows us to use yeast genetics to address these issues and raise hypotheses testable in human cells or small animal models.

## 3. Yeast: A Model to Tackle Complex Purine-Associated Diseases

Metabolic genetic diseases most often affect metabolic enzymes which in the mutant form is either inactive (loss of function), hyperactive or inadequately regulated. These dysfunctions can result in different metabolic consequences which are not at all exclusive:

- Important metabolites may not be synthesized, or synthesized insufficiently
- Metabolites, which are accumulated due to an increased synthesis or the lack of metabolizing enzymes, can be toxic because they interfere with other processes (this could be particularly true for metabolites which have physiological regulatory properties)
- A metabolic unbalance can occur, which is often due to a mix of the first two hypotheses

In many instances, mutations in yeast and human orthologues not only result in the same biochemical dysfunction (typically loss of activity) but also may lead to highly similar secondary consequences. Below, using various examples, we will illustrate the issues raised by complex metabolic diseases and how yeast can help to address these.

### 3.1. AMP-Deaminase (AMPD2) Deficiency Associated with Pontocerebellar Hypoplasia Results in Defective ATP/GTP Balance

AMP-deaminase is an important purine interconversion enzyme, which allows synthesis of IMP from AMP (Figure 1). In yeast there is a unique isoform encoded by the *AMD1* gene, while in humans there are three isoforms expressed in different tissues. Deficiency of the muscular form, *AMPD1*, is associated with hyper-fatigability and is a relatively frequent mutation, although it is often asymptomatic, which suggests complex interplays with other factors [8]. The lack of the yeast enzyme was associated with an increased ATP and low GTP under conditions where ATP was synthesized from adenine. In the meantime, the nucleotide balance was unaffected when *amd1*-deleted yeast cells were grown under conditions where ATP was synthesized from IMP [33]. This conditional phenotype lead to the conclusion that the low GTP was a result of ATP accumulation, which occurred through an ATP feedback inhibition of Ade4, the first enzyme of the de novo pathway [13]. This allosteric inhibition resulted in a lowered IMP synthesis and consequently a low intracellular GMP and GTP [33]. More recently, the identification in humans of *AMPD2* deficiency as the cause of pontocerebellar hypoplasia in a cohort of patients revealed that the exact same phenomenon operated in human cells [34]. These authors showed that, just as in yeast, the GTP shortage in *AMPD2* deficient human cells was dependent on replenishment of the adenylic nucleotide pool by a purine precursor (adenosine for human cells, adenine for yeast) [33,34]. In both cases, ATP accumulation resulted in a low GTP, through regulatory means. Thus, in the AMP deaminase-deficient cells, ATP was toxic by affecting the ATP/GTP balance in both yeast and human cells. Accordingly, restoring intracellular GTP through guanine or AICAR feeding in yeast and human cells, respectively, was sufficient enough to abolish ATP toxicity [33,34]. This illustrated how a single mutation could cause a complex phenotype through shortage of the reaction product (IMP), accumulation of the substrate (AMP) and the resulting imbalance of downstream metabolic products (ATP and GTP).

In their study, Akizu and coworkers also took advantage of yeast to functionally validate *AMPD2* as an AMP deaminase by complementation of the *amd1* growth defect specifically on adenine. They also expressed Human *AMPD2* mutant forms in yeast cells and showed that these alleles resulted in poorly functional enzymes [34]. Finally, these authors identified translation initiation as a defect resulting from GTP shortage, in both humans and yeast, and proposed that it could contribute to the etiology of this disease [34]. Importantly, once again yeast was used to document this phenomenon in depth [34]. This study thus nicely illustrates not only how information derived from yeast genetics can be used to characterize a human disease, but also how yeast can be used as a tool to functionally validate the various alleles of a human gene or to study molecular mechanisms that could cause the disease. The authors proposed that the dependence on adenosine for expression of the phenotype could explain the neural-specificity of the defect, since a significant amount of adenosine was present in the brain [35]. Hence, “brainless” yeast proved to be very useful for understanding this neurodegenerative disorder. More generally, this work exemplifies the remarkable conservation of genes, functions, phenotypes and mechanisms between human and yeast.

### 3.2. Deficiencies in the Purine De Novo Pathway: Toxic Accumulation of Metabolic Intermediates?

Strikingly, while a succession of 13 enzymatic steps are required for AMP de novo synthesis, so far mutations in only three of the corresponding genes have been identified as associated with diseases. These three genes encode phosphoribosyl pyrophosphate synthase (PRPS) [29,30,31,32], adenylosuccinate lyase (ADSL) [36] and AICAR-transformylase IMP cyclohydrolase (ATIC) [25]. The reasons why no disease has ever been associated with mutations in the other steps of the de novo pathway are not known, but it is remarkable that SZMP (Succinyl Amino Imidazole Carboxamide Ribonucleotide monophosphate) and ZMP (Amino Imidazole Carboxamide ribonucleotide monophosphate), the substrates of both ADSL and ATIC respectively (Figure 1), were identified as two major regulator metabolites in yeast [12,13,14]. This suggests that the disease caused by the enzymatic defect could mostly be due to a toxic accumulation of the enzyme-substrates and/or derivatives, rather than by the block in the de novo pathway.

For ADSL, the situation is even more complex as this enzyme acts at two different steps of AMP synthesis and its defect leads to accumulation of its two substrates, SZMP and SAMP (Succinyl AMP). Both metabolites are suspected to contribute to the etiology of the disease, and their relative abundance was proposed to be relevant for symptom severity [37,38], although this assumption is still debated [39,40]. Yeast genetics (our unpublished results) revealed that the *ADSL* knock-out mutants were genetically unstable and tended to pick-up suppressor mutations upstream in the de novo pathway that would block substrate accumulation. Indeed, in yeast, under conditions where the pathway is constitutively turned-on, SZMP and/or SAICAR (Succinyl Amino Imidazole Carboxamide Ribonucleoside) toxicity clearly correlated to the strength of the allele i.e., to the level of residual ADSL activity [14]. The reasons for SZMP/SAICAR toxicity are not elucidated, but could be related to the regulatory roles of this small molecule. In yeast, SZMP promotes an interaction between two transcription factors, Bas1 and Pho2, and thereby stimulates transcription of the purine regulon [12], while in humans, SZMP specifically stimulates PKM2 in cancer cells [11].

ATIC deficiency is a very rare disease that leads to a massive accumulation of AICAR monophosphate (ZMP) [25]. In yeast, ATIC mutants (the *ade17* mutant and *ade16* *ade17* double mutant) massively accumulate ZMP but also the other nucleosides (AICAR (Amino Imidazole Carboxamide ribonucleoside), SAICAR and Succinyl-adenosine) and nucleotide monophosphate derivatives (SZMP and SAMP). We have shown that an enzymatic inhibition and/or reversion of ADSL (Ade13) by ZMP are responsible for the accumulation of these succinyl derivatives in yeast [12,13]. It could be then expected that the accumulation of these derivatives also observed in ATIC patients [25] could be linked to a similar phenomenon. Yeast thus offers genetic and biochemical tools that can help identify some sources of toxicity for those AICAR derivatives. Indeed, by proteomic approaches, we have recently identified yeast proteins that specifically bind ZMP and some of its derivatives (AICAR, SZMP and AMP) [41]. A similar approach was also performed with mammalian proteins (human cells; M. Duperray, M. Moenner and B. Pinson, unpublished results) and revealed 104 human proteins specifically binding the ZMP resin. Among those human proteins, 70 have a yeast ortholog of which one third were previously identified as specific ZMP binders [41]. Molecular studies of the ZMP effect on some of these proteins can provide new insights into the biological defects associated with human ADSL and/or ATIC deficiencies.

Strikingly, AICAR and ZMP also accumulate in other human purine-associated diseases such as in HGPRT deficiency [42,43], and this was also observed in *hpt1* mutant yeast cells [44], suggesting once again a high functional conservation of the metabolic balances in the two organisms. Importantly, a HGPRT deficiency in yeast was synthetically lethal with ZMP accumulation [45], suggesting that the small molecule could contribute to the etiology of the disease. Interestingly, our genetic analyses of yeast revealed mutations in GMP kinase (Guk1, Figure 1) that mimicked the HGPRT deficiency phenotypes: hypoxanthine utilization, purine excretion [23] and also ZMP accumulation [44]. Whether a similar phenocopy mechanism exists in human and which could account for some of the “non-HGPRT-dependent HGPRT-like deficiencies”, remains to be investigated. How and to what extend ZMP accumulation contributes to the pathological symptoms associated with HGPRT-deficiency remains to be established, but this hypothesis has been raised in the past [46].

Interestingly, in yeast, while both ZMP and SZMP are toxic [14], ZMP appeared to be more toxic than SZMP since synthesis of SZMP from ZMP increased AICAR-resistance, while on the other hand blocking synthesis of SZMP from ZMP increased AICAR sensitivity [41]. Beside its potential implication in purine metabolic diseases as a toxic metabolite, AICAR is used as an antiproliferative prodrug. Indeed, AICAR is toxic for tumor cells of multiple origins [47] and in particular to aneuploid cells [48,49] thus raising interesting perspectives as an anticancer molecule. Importantly, it was well tolerated in phase I/II clinical trials [50] and has proved efficient in several different xenograft assays [48,51,52]. Yeast genetics has been used to identify the sources of sensitivity and resistance to AICAR, including uptake [53], carbon utilization [45], nuclear import [41,54] and the ubiquitin pathway [55]. Importantly, based on the results obtained in these yeast studies, we found that AICAR toxicity was increased in the knock-down of human genes (*RNF40*, *ASH2L*, *MLL2*) corresponding to the yeast mutations (*bre1*, *set1*) [54]. Hence yeast, besides being a model for purine metabolic diseases, can also be used to reveal genetic backgrounds specifically affected by drugs, including toxic nucleoside analogs such as AICAR [56] or purine metabolism inhibitors such as mycophenolic acid [57], an IMPDH inhibitor which is used as an immunosuppressant in clinics.

## 4. Yeast as a Tool to Functionally Validate Orthologous Genes from Other Model Organisms

Though yeast studies can be very informative about molecular mechanisms associated with purine metabolism diseases, animal models are required to study tissue specific or developmental aspects that are the most likely determinant in the etiology of purine metabolism diseases. While mice have been used as a model animal, for example to study HGPRT deficiency [58], new models are emerging that are more prone to developmental studies due to external development such as the zebra fish [59] or *Xenopus laevis* (our unpublished work), or more open to the possibility of running genetic experiments (nematode, [60]). Although the genomes of these organisms are fully sequenced, very little is known about their purine metabolism. As a first step it is essential to identify the purine metabolism coding genes (classically by homology search) and most importantly to validate them functionally. This functional validation can be done by complementation of the corresponding knock-out mutants in yeast. Our unpublished work on *X. laevis* and on the nematode *Caenorhabditis elegans* allowed such a functional validation for most of the candidate purine metabolism genes via their expression in yeast. As an illustration, the *paics1.L* *X. laevis* gene restored growth of an *ade1* knock-out yeast mutant in the absence of external purine (Figure 2 and our unpublished results) or for *C. elegans* gene candidates [60]. Yeast can also be used for in vivo studies of the effects of mutations in human genes, as was done for example for APRT [61]. Hence, yeast not only proved useful as a model by itself but also to help set up new models more disposed to addressing specific questions such as tissue specificity or developmental aspects. Taking advantage of the complementarity of various models has been a fruitful strategy for tackling highly integrated biological questions, and for providing some understanding about complex human diseases. In this respect, yeast has very efficiently played its part, and will certainly continue to help us address challenging questions in the future.

## 5. Conclusions

It is striking that the purine de novo pathway is highly conserved between yeast and human (although some enzymes are much more divergent than others), while the salvage pathway shows much more differences between these species (Figure 1). However, despite these specific enzyme differences, several examples reported here reveal a very strong functional conservation, hence highlighting that similar mechanisms ensure homeostasis in both organisms. This functional conservation operates both at the metabolic molecular level, i.e., purine nucleotide content and balance, but also more widely at the cellular or organism level, as revealed by phenotypical outcomes. Due to this highly functional conservation, yeast, which is prone to genetics, proteomic and metabolic studies, provides an interesting model to elaborate new working hypotheses testable in human cells or animal models. In particular, because of the high redundancy of metabolic pathways, combinations of mutants are often required to reveal phenotypes, and while the construction of double or triple mutants is trivial in yeast, it is still challenging in most eukaryote organisms.

## Figures and Tables

**Table 1:** Pathologies associated with defects in purine synthesis enzymes highly conserved between yeast and humans.

Pathology	Enzymatic Defect	Gene Name (Location)	Phenotype MIM Number	Inheritance	Locus MIM Number	Reference
ADSL deficiency	Loss of function	*ADSL* (22q13.1)	103050	Autosomal recessive	608222	[24]
AICA-Ribosiduria	Loss of function	*ATIC* (2q35)	608688	Autosomal recessive	601731	[25]
Retinitis pigmentosa 10	Loss of function	*IMPDH1* (7q32.1)	180105	Autosomal dominant		[26]
Leber Congenital Amaurosis 11	Loss of function	*IMPDH1* (7q32.1)	613837	?	146690	[27]
PNP deficiency		*PNP* (14q11.2)	613179	Autosomal recessive	164050	[28]
Arts syndrome	Loss of function	*PRPS1* (Xq22.3)	311835	X-linked recessive	311850	[29]
Charcot-Marie-Tooth disease, X-linked recessive, 5	Loss of function	*PRPS1* (Xq22.3)	311070	X-linked recessive	311850	[30]
Deafness, X-linked 1	Loss of function	*PRPS1* (Xq22.3)	304500	X-linked	311850	[31]
Hyperuricemia, PRPS-related	Gain of function	*PRPS1* (Xq22.3)	300661	X-linked recessive	311850	[32]

## Appendix A

**Table A1:** List of the *Saccharomyces cerevisiae* purine pathway enzymes shown in Figure 1. Enzyme features were obtained from the *Saccharomyces* Genome Database website [62].

Protein Name	ORF Name	Enzymatic Activities
Aah1	*YNL141W*	Adenine deaminase
Ade1	*YAR015W*	Phosphoribosyl aminoimidazole succinocarboxamide synthetase
Ade12	*YNL220W*	Adenylosuccinate synthase
Ade13	*YLR359W*	Adenylosuccinate lyase
Ade16	*YLR028C*	AICAR transformylase and IMP cyclohydrolase
Ade17	*YMR120C*	AICAR transformylase and IMP cyclohydrolase
Ade2	*YOR128C*	Phosphoribosylaminoimidazole carboxylase
Ade4	*YMR300C*	Phosphoribosylpyrophosphate amidotransferase
Ade5,7	*YGL234W*	Aminoimidazole ribonucleotide synthetase and glycinamide ribotide synthetase
Ade6	*YGR061C*	Formylglycinamidine-ribonucleotide synthetase
Ade8	*YDR408C*	Phosphoribosyl-glycinamide transformylase
Adk1	*YDR226W*	Adenylate kinase
Adk2	*YER170W*	Adenylate kinase
Ado1	*YJR105W*	Adenosine kinase
Amd1	*YML035C*	AMP deaminase
Apt1	*YML022W*	Adenine phosphoribosyltransferase
Gua1	*YMR217W*	GMP synthase
Gud1	*YDL238C*	Guanine deaminase
Guk1	*YDR454C*	Guanylate kinase
Hpt1	*YDR399W*	Hypoxanthine-guanine phosphoribosyltransferase
Imd2	*YHR216W*	Inosine monophosphate dehydrogenase
Imd3	*YLR432W*	Inosine monophosphate dehydrogenase
Imd4	*YML056C*	Inosine monophosphate dehydrogenase
Isn1	*YOR155C*	Inosine monophosphate specific nucleotidase
Phm8	*YER037W*	GMP, UMP and CMP nucleotidase
Pnp1	*YLR209C*	Purine nucleoside phosphorylase
Prs1	*YKL181W*	PRPP synthetase subunit
Prs2	*YER099C*	PRPP synthetase subunit
Prs3	*YHL011C*	PRPP synthetase subunit
Prs4	*YBL068W*	PRPP synthetase subunit
Prs5	*YOL061W*	PRPP synthetase subunit
Rnr1	*YER070W*	Large subunit of ribonucleotide-diphosphate reductase
Rnr2	*YJL026W*	Small subunit of ribonucleotide-diphosphate reductase
Rnr3	*YIL066C*	Large subunit of ribonucleotide-diphosphate reductase
Rnr4	*YGR180C*	Small subunit of ribonucleotide-diphosphate reductase
Ynk1	*YKL067W*	Nucleoside diphosphate kinase

**Table A2:** List of the human purine pathway enzymes shown in Figure 1. Enzyme features were obtained from the NCBI web site [63].

Protein Name	Gene Location	Enzymatic Activity
ADA	20q13.12	Adenosine deaminase
ADK	10q22	Adenosine kinase
ADSL	22q13.1	Adenylosuccinate lyase
ADSS	1q44	Adenylosuccinate synthase
AK1	9q34.11	Adenylate kinase
AK2	1p35.1	Adenylate kinase
AK3	9p24.1	Adenylate kinase
AK4	1p31.3	Adenylate kinase
AK5	1p31.1	Adenylate kinase
AK6	5q13.2	Adenylate kinase
AK7	14q32.2	Adenylate kinase
AK8	9q34.13	Adenylate kinase
AK9	6q21	Adenylate kinase
AMPD1	1p13.2	Adenosine monophosphate deaminase
AMPD2	1p13.3	Adenosine monophosphate deaminase
AMPD3	11p15.4	Adenosine monophosphate deaminase
APRT	16q24.3	Adenine phosphoribosyltransferase
ATIC	2q35	Amino-Imidazole CarboxAmide Ribonucleotide transformylase and IMP cyclohydrolase
GAH	9q21.13	Guanine deaminase
GART	21q22.11	Phosphoribosylglycinamide formyltransferase, phosphoribosylglycinamide synthetase and phosphoribosylaminoimidazole synthetase
GMPS	3q25.31	Guanine monophosphate synthase
GUK1	1q42.13	Guanylate kinase
GUK2	1q32.1-q42	Guanylate kinase
HPRT1	Xq26.1	Hypoxanthine phosphoribosyltransferase
IMPDH1	7q32.1	Inosine monophosphate dehydrogenase
IMPDH2	3p21.31	Inosine monophosphate dehydrogenase
NME1	17q21.33	Nucleoside diphosphate kinase
NME2	17q21.33	Nucleoside diphosphate kinase
NME3	16p13.3	Nucleoside diphosphate kinase
NME4	16p13.3	Nucleoside diphosphate kinase
NME5	5q31.2	Nucleoside diphosphate kinase
NME6	3p21.31	Nucleoside diphosphate kinase
NME7	1q24.2	Nucleoside diphosphate kinase
PAICS	4q12	Phosphoribosylaminoimidazole carboxylase and phosphoribosylaminoimidazolesuccinocarboxamide synthetase
PFAS	17p13.1	Phosphoribosylformylglycinamidine synthase
PNP	14q11.2	Purine nucleoside phosphorylase
PPAT	4q12	Phosphoribosyl pyrophosphate amidotransferase
PRPS1	Xq22.3	Phosphoribosyl pyrophosphate synthetase 1
PRPS2	Xp22.2	Phosphoribosyl pyrophosphate synthetase 2
RRM1	11p15.4	Ribonucleotide reductase catalytic subunit
RRM2	2p25.1	Ribonucleotide reductase regulatory subunit
RRM2B	8q22.3	Ribonucleotide reductase regulatory subunit
XDH	2p23.1	Xanthine dehydrogenase/oxidase

**Table A3:** Comparison of the yeast and human purine pathway enzymes depicted in Figure 1. Identity and similarity scores were obtained from the Proteome^TM^ platform [64]. For each enzymatic activity, only the best score obtained between yeast and a human ortholog was shown. Protein coverage refers to the fraction (%) of the entire protein (the smallest of the two orthologs) used in the alignment for determination of identity and similarity scores.

Yeast Protein	Human Protein	Identity (%)	Similarity (%)	Protein Coverage (%)
Rnr2	RMM2	69	81	90
Rnr3	RMM1	66	84	87
Ade13	ADSL	64	79	97
Prs3	PRPS2	61	78	79
Ynk1	NME2	61	80	97
Imd2	IMPD2	61	78	98
Prs2	PRPS1	60	75	78
Ade16	ATIC	60	75	99
Ade17	ATIC	60	75	99
Adk1	AK2	58	73	98
Ade12	ADSS	52	68	99
Pnp1	PNP	51	68	85
Guk1	GUK1	51	70	99
Amd1	AMPD2	50	66	74
Ade5,7	GART	45	64	99
Apt1	APRT	44	64	96
Gud1	GAH	42	49	99
Ado1	ADK1	36	57	99
Gua1	GMPS	36	54	100
Ade4	ATASE/PPAT	34	51	92
Ade6	PFAS	33	49	99
Hpt1	HGPRT	33	46	33
Ade8	GART	32	52	92
Ade2	PAICS	27	45	99
Ade1	PAICS	22	41	100
